# Longitudinal spatial changes of the contralesional corticospinal tract in high-grade glioma: a within-patient DTI tractography study

**DOI:** 10.1007/s11060-026-05823-w

**Published:** 2026-10-09

**Authors:** Bingzhen Li, Raimunde Liang, Ghaith Altawalbeh, Lisa Hoenikl, Eugen Ursu, Axel Schroeder, Haosu Zhang, Philipp Karschnia, Chiara Negwer, Bernhard Meyer, Arthur Wagner

**Affiliations:** 1 Department of Neurosurgery, Hospital of the Technical University Munich, Munich, Germany; 2 Department of Neurosurgery, Kantonsspital St. Gallen, Medical School of St. Gallen, University of St. Gallen, St. Gallen, Switzerland; 3 Department of Neurosurgery, Tiantan Hospital (Anhui Campus), Beijing Medical University, Wuhu, Anhui China

**Keywords:** Glioma, Corticospinal tract, Contralesional hemisphere, Diffusion tensor imaging, Tractography, Longitudinal analysis

## Abstract

**Purpose:**

The contralesional corticospinal tract (CST) is often used as an internal reference in glioma tractography, but its longitudinal stability remains poorly understood. We explored within-patient changes in contralesional CST characteristics between primary and recurrent high-grade glioma.

**Methods:**

Twenty-nine patients with paired preoperative diffusion tensor imaging at primary and recurrent assessments were retrospectively analyzed. The contralesional CST was reconstructed using anatomically guided deterministic tractography at fractional anisotropy thresholds (FATs) of 0.10 and 0.15. Tract-averaged fractional anisotropy (FA), tract length, and tract volume were compared longitudinally. Spatial changes were assessed using patient-level slice-wise centroid trajectories and paired voxel-wise analyses in standardized space, with HCP842 used as a fixed normative spatial reference.

**Results:**

No longitudinal differences in FA, tract length, or tract volume remained significant after BH-FDR correction. In contrast, slice-wise analyses showed a predominantly anterior CST displacement at recurrence, with mean paired Y-axis differences of 2.576 mm at FAT 0.10 and 2.078 mm at FAT 0.15; 57 and 21 slices, respectively, remained significant after correction. Mediolateral changes were smaller and less consistent, and no voxel-wise differences survived correction. Relative to HCP842, both assessments were posterior to the reference trajectory, while the recurrent CST was less posterior.

**Conclusion:**

The contralesional CST showed longitudinal spatial displacement without corresponding corrected changes in tractography-derived FA, tract length, or tract volume. These findings indicate that its spatial position may not remain fixed between primary and recurrent glioma assessments.

**Supplementary Information:**

The online version contains supplementary material available at https://doi.org/10.1007/s11060-026-05823-w.

## Introduction

Preservation of functionally relevant neural structures is a central consideration in neurosurgical oncology and may require adaptation of the anatomical relationship between the tumor and surrounding neural structures [1]. Gliomas involving motor-related regions can alter the corticospinal tract (CST) through displacement, infiltration, disruption, and diffusion changes. DTI-based tractography is widely used to delineate the CST and characterize its spatial relationship to the tumor preoperatively [2, 3]. Recent studies have refined CST reconstruction by integrating anatomical and functional information [4]. Most previous work, however, has focused on the tumor-bearing hemisphere.

The contralesional CST is frequently used as a patient-specific comparator because it is remote from the lesion and reduces interindividual variability [5, 6]. This approach assumes relative stability of the contralesional tract. However, diffusion abnormalities have been reported in contralateral normal-appearing white matter in malignant glioma [7, 8], and serial DTI has demonstrated white-matter changes during treatment for high-grade glioma [9, 10]. Whether the contralesional CST itself changes between primary and recurrent disease remains unclear.

Such changes may involve quantitative tractography-derived characteristics, spatial organization, or both. Global tract measures and tract position describe different features of the reconstructed CST. If the contralesional CST changes longitudinally, its use as a stable reference in serial glioma studies may require caution. Given limited glioma-specific evidence, the study was exploratory, with no prespecified directional change.

We therefore performed a longitudinal within-patient study of the contralesional CST in primary and recurrent high-grade glioma, evaluating FA, tract length, tract volume, spatial trajectory, and voxel-wise distribution in standardized space.

## Materials and methods

### Study design, ethics, and patient cohort

This retrospective single-center longitudinal study included 29 adults treated between 2013 and 2021. Eligibility required (1) frontal-lobe high-grade glioma; (2) documented first recurrence based on clinical imaging assessment; (3) preoperative DTI at both assessments; and (4) MRC strength of 5/5 at both assessments. Motor function was assessed clinically using the MRC scale; MRC 5/5 at both assessments was required to minimize potential confounding from overt motor deficits and to focus the analysis on patients with preserved motor strength. Patients were excluded for incomplete or artifact-degraded imaging, failed contralesional CST reconstruction at either assessment, or failed spatial-normalization quality control.

Clinical data included age, sex, pathological grade, IDH status, MGMT promoter status, treatment between assessments, and the imaging interval. Age at the primary assessment was estimated from birth year when complete birth dates were unavailable. Tumor volume and minimum tumor-to-precentral-gyrus distance were measured in Brainlab at both assessments.

The study was approved by the local ethics committee (registration no. 2026-402-S-CT) and conducted in accordance with the Declaration of Helsinki; informed consent was waived because of the retrospective design.

### MRI acquisition

At both assessments, preoperative MRI was performed 1–7 days before surgery on a 3-T scanner (Achieva 3 T, Philips Medical Systems, Best, the Netherlands). Contrast-enhanced T1-weighted images were acquired with a gradient-echo sequence (TR/TE, 9/4 ms; 1-mm isotropic voxels) after intravenous gadolinium administration. DTI was acquired in the same session using 32 diffusion directions (b = 1000 s/mm²; TR/TE, 5000/78 ms; 2-mm isotropic voxels).

### Contralesional CST reconstruction and quantitative analysis

Before fiber tracking, the T1-weighted and diffusion datasets were co-registered within Brainlab Elements and processed using the software-integrated diffusion-tensor pipeline. The contralesional CST was reconstructed using deterministic DTI-based fiber tracking with the same anatomically guided protocol at both assessments. A deep ROI was manually delineated at the cerebral peduncle/brainstem level on the axial section showing its largest cross-sectional extent. ROI placement was standardized by anatomical level and landmarks, whereas ROI size was adapted to individual anatomy to encompass the relevant descending fibers. The internal capsule served as an anatomical constraint and the precentral gyrus as the cortical target.

Tracking was performed at FATs of 0.10 and 0.15, with a minimum fiber length of 100 mm and a maximum turning angle of 20°. Reconstructions were visually inspected, and anatomically implausible streamlines were removed when necessary. The reconstructed pathway represented the tractography-defined motor portion of the CST rather than the entire anatomical CST. Tract-averaged FA, tract length, and tract volume were obtained directly from Brainlab for longitudinal comparison.

### Spatial normalization and side harmonization

Reconstructed CSTs were converted to binary NIfTI masks for spatial analysis. At each assessment, the contrast-enhanced T1-weighted image was normalized to MNI152 space using SPM12 (Normalize: Estimate and Write), and the corresponding CST mask was transformed with the same deformation field using nearest-neighbor interpolation, yielding a common 157 × 189 × 156 grid with 1-mm isotropic voxels. Transformed CST masks were then re-binarized at values > 0.

Normalized T1-weighted images were visually inspected against the MNI152 template, and transformed CST masks were checked for alignment with the corresponding normalized T1-weighted images. Grid dimensions, affine information, and primary–recurrence pairing were also verified.

For side harmonization, all contralesional CSTs were oriented to the negative MNI-X side. When reflection was required, primary and recurrent masks from the same patient were reflected together to preserve within-patient spatial correspondence; 12 of 29 patient pairs required reflection.

### Slice-wise and voxel-wise spatial analysis

For each patient, assessment, FAT, and axial slice, centroids of nonzero CST voxels were calculated in MNI X and Y coordinates. After side harmonization, X represented the mediolateral direction and Y the anteroposterior direction. Longitudinal displacement was defined as recurrent minus primary; positive ΔX and ΔY indicated medial and anterior displacement, respectively. Slice-wise analyses included only axial levels with complete centroid data from all 29 patient pairs at both assessments. Patient-level X and Y centroids were compared between assessments at each eligible slice, and mean recurrence-minus-primary differences across the eligible range were summarized descriptively. Pooled-voxel trajectories were generated for visualization by combining CST voxels across patients within each assessment and calculating slice-wise centroids. Because this approach does not preserve equal patient weighting, pooled trajectories were used for descriptive visualization only.

For voxel-wise analysis, primary and recurrent binary CST masks were compared at each voxel across the 29 matched pairs using the exact McNemar test. Testing was restricted to the union of voxels occupied at either assessment within each FAT. BH-FDR and Bonferroni correction were applied across all tested voxels. Uncorrected *P* < 0.05 maps were additionally displayed after a minimum cluster-size filter of 20 contiguous voxels and were considered descriptive rather than statistically corrected.

### Normative-reference and exploratory bilateral analyses

The HCP842 CST atlas [11] was resampled to the same MNI grid and harmonized to the analyzed side. Patient-minus-HCP842 X- and Y-centroid offsets were calculated for each patient and assessment across the eligible slice range and summarized across all 29 patients with 95% confidence intervals. Because HCP842 is a fixed normative reference generated using a different diffusion acquisition and tractography pipeline, these comparisons were descriptive rather than healthy-control inference.

An exploratory bilateral analysis included 13 patients with tumor-side and contralesional CST masks at both assessments and FATs. Tumor-side CSTs used nTMS-based motor cortical seeding, whereas contralesional CSTs used anatomical cortical seeding. All masks were harmonized to the same MNI-X side. Patient-level tumor-side-minus-contralesional X- and Y-centroid differences were calculated at each assessment, with the side-by-time interaction defined as their change from primary to recurrence. Because seeding strategies differed between sides, this analysis was exploratory, and no biological inference was based on volume or voxel-count asymmetry.

### Statistical analysis

Statistical analyses were performed in Python 3.12.3 (Anaconda). Continuous variables are reported as mean ± standard deviation or median [interquartile range], and categorical variables as n (%). Tumor volume was compared using the Wilcoxon signed-rank test and tumor-to-precentral-gyrus distance using a paired t test. Because all primary–recurrent comparisons were performed within the same patients, sex and baseline age were not included as covariates in the primary analyses.

Tract-averaged FA, tract length, and tract volume were compared between assessments using paired t tests at each FAT, with 95% confidence intervals and Cohen’s dz effect sizes; Wilcoxon signed-rank tests were used as sensitivity analyses. BH-FDR correction was applied across the six prespecified comparisons (three measures × two FATs), with sensitivity analyses corrected separately.

**Fig. 1:**
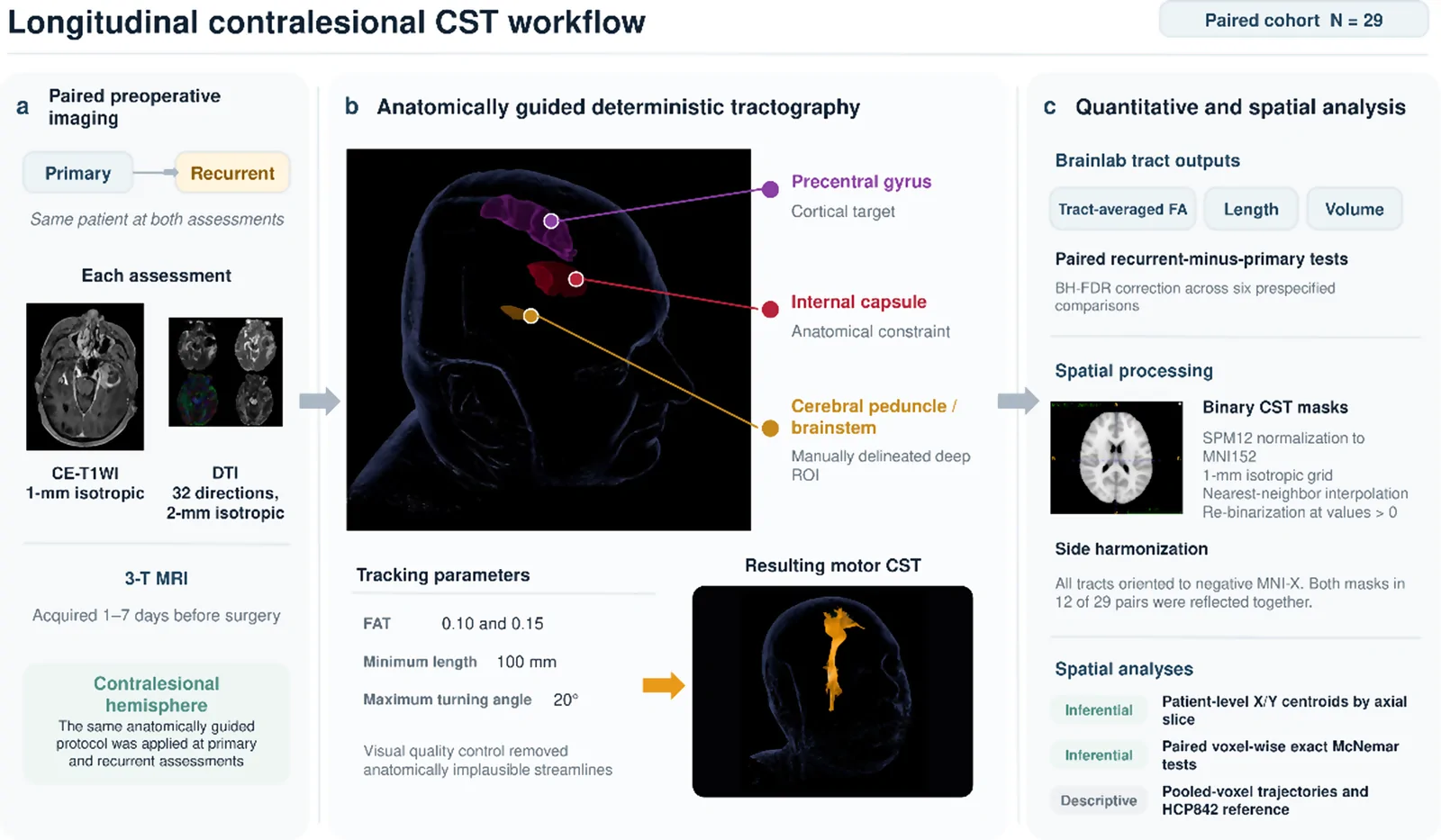
Overall longitudinal workflow for contralesional CST analysis. (**a**) Paired preoperative CE-T1WI and DTI were obtained at the primary and recurrent assessments in the same patients. (**b**) The contralesional CST was reconstructed using anatomically guided deterministic DTI-based tractography. The precentral gyrus served as the cortical target, the internal capsule as an anatomical constraint, and a manually delineated ROI at the cerebral peduncle/brainstem level as the deep ROI. Tracking was performed at FATs of 0.10 and 0.15, with a minimum fiber length of 100 mm and a maximum turning angle of 20°.(**c**) Tract-averaged FA, tract length, and tract volume were obtained from Brainlab. Binary CST masks were normalized to MNI152 space using the same deformation field, transformed with nearest-neighbor interpolation, re-binarized, and harmonized to the same MNI-X side before slice-wise centroid analysis, paired voxel-wise exact McNemar testing, and descriptive comparison with the HCP842 normative reference

Slice-wise X- and Y-centroid differences were analyzed using paired t tests with Wilcoxon sensitivity analyses. Within each FAT, BH-FDR correction was applied jointly across all eligible X- and Y-axis paired t tests; Wilcoxon sensitivity tests were corrected separately using the same procedure. Voxel-wise exact McNemar tests used BH-FDR and Bonferroni correction within each FAT, and exploratory bilateral analyses used BH-FDR correction across six global tests per FAT. All tests were two-sided; raw P and BH-FDR-corrected q values are reported, with q < 0.05 considered significant. The overall workflow is shown in Fig. 1.

## Results

### Demographic, clinical, molecular, and treatment characteristics

The cohort comprised 29 patients with paired primary and recurrent assessments (Table 1), with a mean age of 47.8 ± 13.0 years; 19 (65.5%) were men. All tumors were frontal and WHO grade 4, and all patients had MRC 5/5 strength at both assessments. IDH status was available in 27 patients, all of whom were IDH wild-type; IDH status was unavailable in the remaining two patients. MGMT promoter methylation was present in 11 patients (37.9%).

Between assessments, 23 patients (79.3%) received radiotherapy-containing treatment, 22 (75.9%) temozolomide-containing treatment, and 19 (65.5%) combined radiochemotherapy or a Stupp regimen. The median assessment interval was 10.3 months [6.0–14.9]. Neither tumor volume (6.19 [2.15–11.40] vs. 2.07 [1.32–3.85] cm³, *P* = 0.121) nor tumor-to-precentral-gyrus distance (42.18 ± 17.46 vs. 43.04 ± 21.42 mm, *P* = 0.758) differed significantly between assessments.

**Table 1:** Demographic, clinical, treatment, and tumor characteristics of the longitudinal cohort

Characteristic		Overall cohort (*N* = 29)		Primary assessment		Recurrent assessment	*P* value
**Demographic and clinical characteristics**							
Patients, n		29					
Age, years		47.8 ± 13.0					
Sex, n (%)							
Male		19 (65.5%)					
Female		10 (34.5%)					
Frontal-lobe tumor, n (%)		29 (100%)					
WHO grade 4, n (%)		29 (100%)					
MRC grade 5/5 at both assessments, n (%)		29 (100%)					
**Molecular characteristics**							
**IDH status**,** n/N (%)**							
IDH wild-type		27/27 (100%)					
IDH unavailable		2					
MGMT promoter status, n (%)							
MGMT methylated		11 (37.9%)					
MGMT unmethylated		18 (62.1%)					
**Longitudinal clinical context**							
Interval between assessments, months		10.3 [6.0–14.9]					
Radiotherapy-containing treatment		23 (79.3%)					
Temozolomide-containing treatment		22 (75.9%)					
Combined radiochemotherapy or a Stupp regimen		19 (65.5%)					
Tumor volume, cm3					6.19 [2.15–11.40]	2.07 [1.32–3.85]	0.121
Tumor–precentral gyrus distance, mm					42.18 ± 17.46	43.04 ± 21.42	0.758

Note: Data are presented as n (%), mean ± standard deviation, or median [interquartile range], as appropriate. Age refers to age at the primary assessment. IDH percentages were calculated among patients with available IDH data (*n* = 27), with unavailable cases reported separately. Treatment categories refer to therapies received between the primary and recurrent assessments and are not mutually exclusive. Primary–recurrent comparisons were performed using paired tests: the Wilcoxon signed-rank test for tumor volume and the paired t-test for tumor–precentral gyrus distance. All tests were two-sided. IDH, isocitrate dehydrogenase; MGMT, O6-methylguanine-DNA methyltransferase; MRC, Medical Research Council

### Longitudinal comparison of tractography-derived CST parameters

None of the six longitudinal comparisons of FA, tract length, or tract volume remained significant after BH-FDR correction (Table 2). At FAT 0.10, no parameter differed significantly between assessments. At FAT 0.15, tract volume was lower at recurrence before correction (raw *P* = 0.041) but did not remain significant after BH-FDR correction (q = 0.244). The Wilcoxon sensitivity analysis yielded the same inference (raw *P* = 0.048; q = 0.288). The paired individual distributions underlying these comparisons are shown in Supplementary Fig. S5 and demonstrate substantial interpatient variability and overlap between the primary and recurrent assessments.

**Table 2:** Paired longitudinal comparison of contralesional CST tractography-derived parameters

FAT	Parameter	Primary assessment	Recurrent assessment	Paired difference† (95% CI)	*P* value	BH-FDR q	t (28)	Cohen’s dz
0.10	Mean FA	0.4676 ± 0.0338	0.466 ± 0.043	−0.002(− 0.018 to 0.014)	0.792	0.793	−0.266	−0.049
	Length, mm	130.34 ± 10.09	132.69 ± 11.46	2.34 (− 1.59 to 6.28)	0.233	0.466	1.219	0.226
	Volume, cm3	16.24 ± 6.51	14.32 ± 5.38	−1.92 (− 4.96 to 1.12)	0.206	0.466	−1.294	−0.240
0.15	Mean FA	0.477 ± 0.041	0.480 ± 0.040	0.003 (− 0.014 to 0.020)	0.712	0.793	0.372	0.069
	Length, mm	126.45 ± 11.58	128.07 ± 13.04	1.62 (− 3.11 to 6.35)	0.489	0.733	0.702	0.130
	Volume, cm3	12.50 ± 5.41	9.90 ± 4.32	−2.60 (− 5.07 to − 0.12)	0.041	0.244	−2.146	−0.399

Data are presented as mean ± SD. † Paired differences were calculated as recurrent minus primary assessment. t statistics are from paired t tests with 28 degrees of freedom, and Cohen’s dz represents the paired-sample effect size. BH-FDR correction was applied across the six prespecified comparisons. The uncorrected reduction in tract volume at FAT 0.15 did not remain significant after correction

### Longitudinal changes in contralesional CST spatial trajectory

**Fig. 2:**
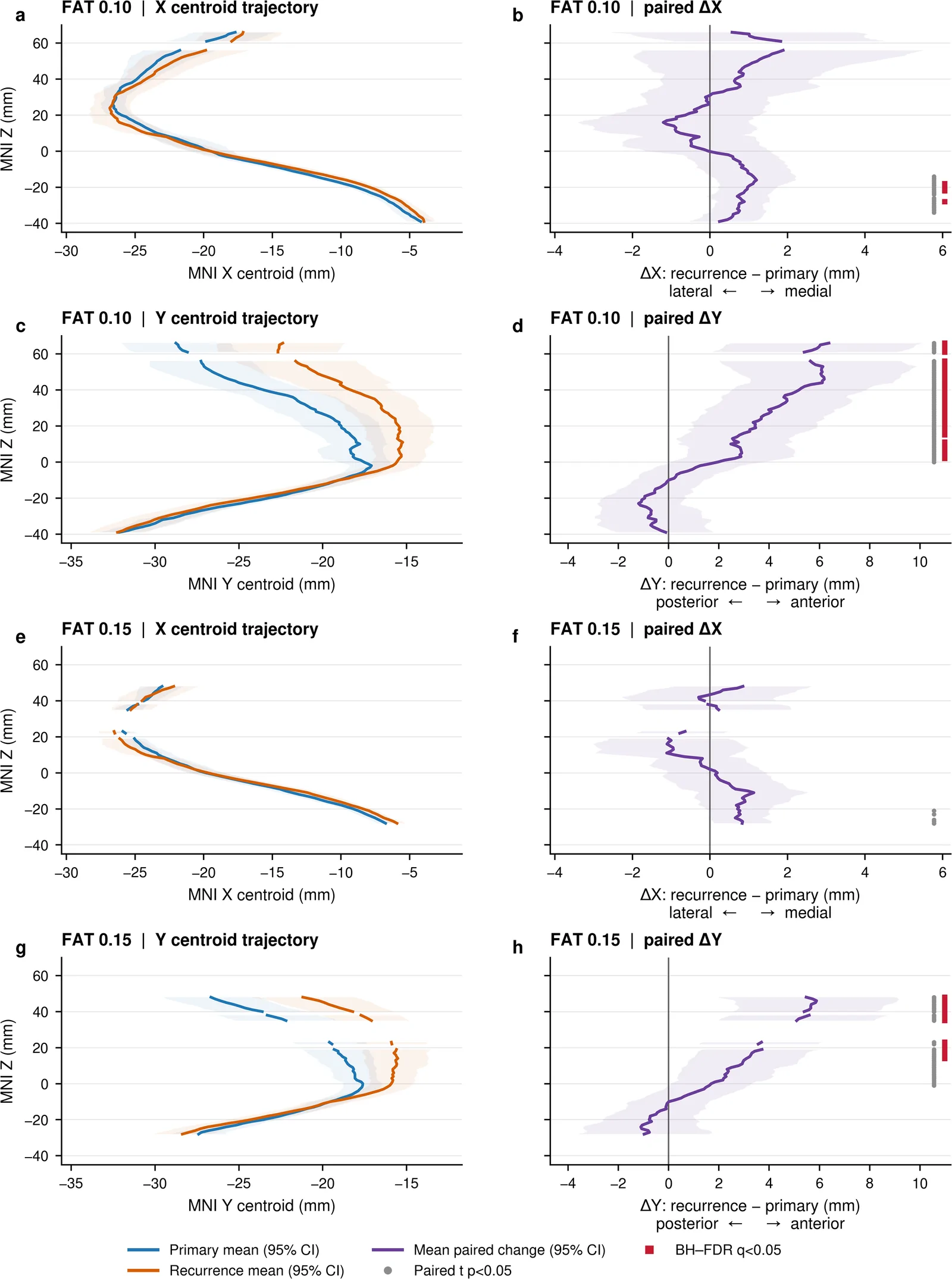
Longitudinal slice-wise changes in contralesional CST position between the primary and recurrent assessments. (**a–d**) FAT 0.10 and (**e–h**) FAT 0.15 analyses. Left panels show mean X- and Y-centroid trajectories across MNI Z levels at the primary and recurrent assessments, with shaded 95% confidence intervals. Right panels show the corresponding mean paired recurrence-minus-primary differences with 95% confidence intervals. Positive ΔX values indicate medial displacement and positive ΔY values indicate anterior displacement. Gray markers denote slices with paired *P* < 0.05, and red markers denote slices remaining significant after BH-FDR correction (q < 0.05). Only slices meeting the prespecified eligibility criteria were included in the slice-wise analyses

Patient-level slice-wise analyses showed a predominantly anterior recurrence-minus-primary displacement along the Y axis (Fig. 2). At FAT 0.10, the mean paired Y-axis difference was 2.576 mm across 102 eligible slices, with 57 slices remaining significant after BH-FDR correction (minimum q = 0.00884). At FAT 0.15, the corresponding difference was 2.078 mm across 63 slices, with 21 corrected slices (minimum q = 0.0143). Wilcoxon sensitivity analyses showed the same pattern, with 39 and 27 corrected slices at FAT 0.10 and 0.15, respectively.

Mediolateral changes were smaller. At FAT 0.10, the mean paired X-axis difference was 0.446 mm medially, with six slices surviving BH-FDR correction but none in the Wilcoxon analysis; at FAT 0.15, the mean difference was 0.124 mm medially, with no corrected slices. Pooled-voxel trajectories similarly showed greater displacement along Y than X, while individual trajectories showed substantial interpatient variability (Supplementary Fig. S2).

No voxel survived BH-FDR or Bonferroni correction in the paired voxel-wise exact McNemar analysis at either FAT (Supplementary Fig. S1); uncorrected cluster-filtered maps were therefore considered exploratory. In the exploratory bilateral analysis of 13 patients, no side-by-time interaction survived global BH-FDR correction (Supplementary Fig. S3). At FAT 0.15, a recurrent tumor-side-minus-contralesional Y offset of 4.453 mm anteriorly was observed (95% CI, 2.077–6.830; q = 0.009), but interpretation was limited by the different cortical seeding strategies used for the two sides.

### Contralesional CST relative to the HCP842 normative reference

**Fig. 3:**
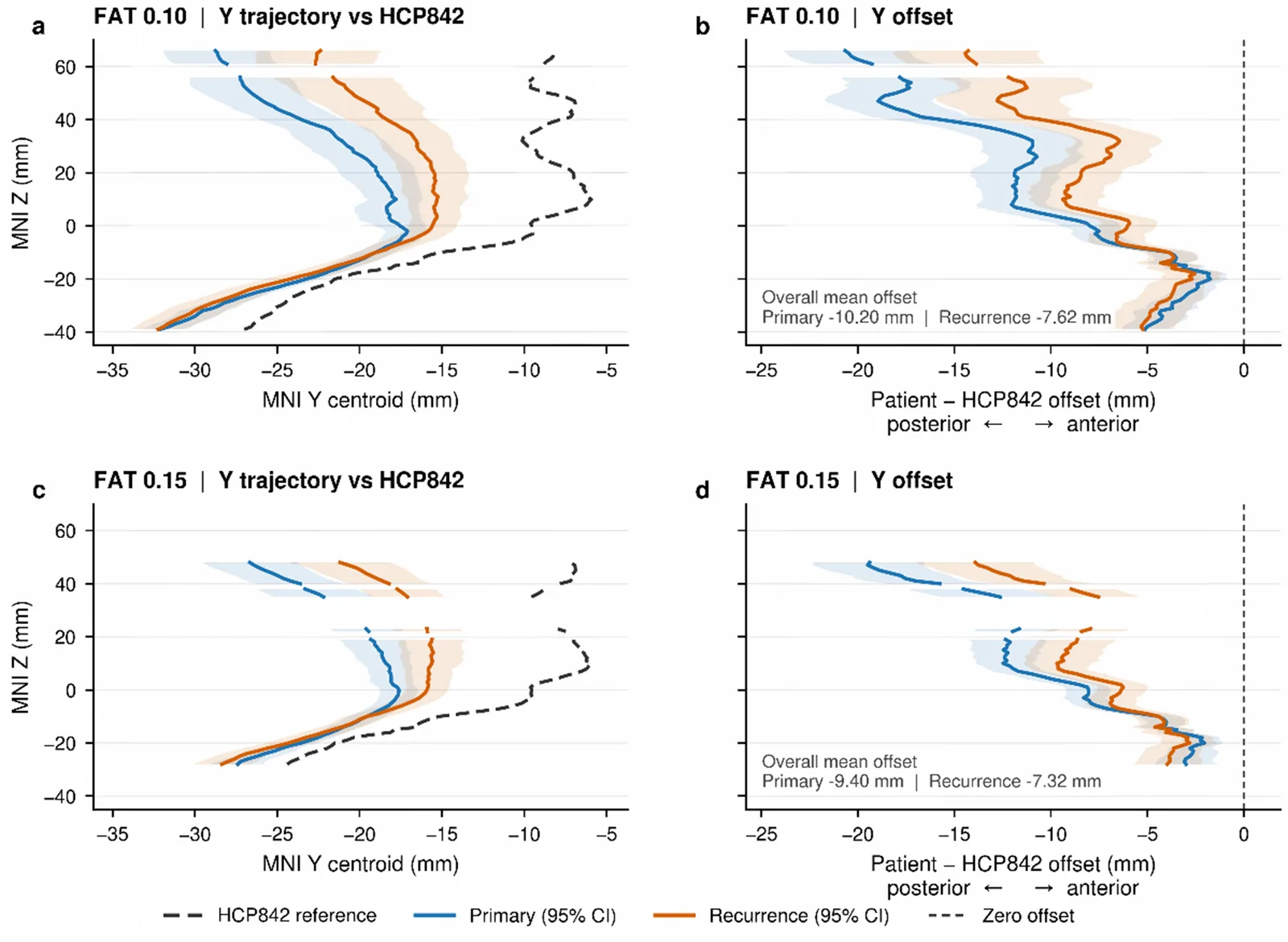
Contralesional CST trajectory relative to the HCP842 normative reference. (a, b) FAT 0.10 and (c, d) FAT 0.15 analyses. Panels a and c show mean Y-centroid trajectories at the primary and recurrent assessments together with the fixed HCP842 reference. Panels b and d show the corresponding patient-minus-HCP842 Y offsets across MNI Z levels. Shaded areas indicate 95% confidence intervals for the patient trajectories, and the dashed vertical line denotes zero offset. Negative offsets indicate a position posterior to the HCP842 reference. Comparisons with HCP842 were descriptive because the atlas represents a single fixed normative reference rather than an independent control cohort

Both primary and recurrent contralesional CST trajectories were posterior to HCP842 along the Y axis (Fig. 3). Mean patient-minus-HCP842 offsets were − 10.20 and − 7.62 mm at FAT 0.10 and − 9.40 and − 7.32 mm at FAT 0.15 for the primary and recurrent assessments, respectively. The recurrent CST was therefore less posterior than the primary CST, consistent with the paired anterior displacement. Comparisons with HCP842 were descriptive because it represents a fixed normative reference rather than an independent control cohort; X-axis results are shown in Supplementary Fig. S4.

## Discussion

In this within-patient longitudinal study, the contralesional CST showed a predominantly anterior shift between the primary and recurrent assessments, whereas FA, tract length, and tract volume showed no significant longitudinal differences after multiple-comparison correction. Mediolateral changes were smaller and less consistent, and no voxel-wise differences survived correction. These findings suggest that contralesional CST position may change longitudinally even when tractography-derived quantitative measures remain stable.

### Longitudinal spatial changes in the contralesional CST

The anterior displacement of the contralesional CST extended across multiple axial levels and was observed at both tracking thresholds, although the spatial extent of significant differences varied between FAT 0.10 and 0.15. Individual trajectories showed substantial variation in both magnitude and direction, indicating that the group-level anterior shift was superimposed on considerable patient-specific variability.

Spatial alterations of white-matter pathways have been described in glioma. DTI studies have demonstrated tumor-associated displacement and changes in tract orientation [12]. More broadly, displacement, infiltration, disruption, and edema-related alterations represent established patterns of glioma-related white-matter involvement [13]. Diffusion abnormalities have also been reported in contralateral normal-appearing white matter in patients with malignant glioma [7, 8]. In addition, serial DTI has demonstrated longitudinal changes in normal-appearing white matter during treatment for high-grade glioma [9]. The present paired analysis provides longitudinal evidence that the spatial trajectory of the contralesional CST may change between the primary and recurrent assessments.

The discrepancy between the slice-wise and voxel-wise results likely reflects the different spatial information captured by the two approaches. Centroid analysis is sensitive to distributed shifts in tract position, whereas voxel-wise McNemar testing requires changes to recur at corresponding voxels across patients. Spatially heterogeneous displacement may therefore alter centroid trajectories without producing a reproducible voxel-wise pattern at the group level.

### Longitudinal stability of quantitative CST measures

In contrast to the spatial changes, FA, tract length, and tract volume showed no significant longitudinal differences after correction for multiple comparisons. The isolated reduction in tract volume at FAT 0.15 did not survive BH-FDR correction and was not reproduced at FAT 0.10. Thus, the longitudinal spatial change was not accompanied by consistent changes in the quantitative characteristics of the reconstructed tract.

These measures should be interpreted as tractography-derived characteristics rather than direct measures of axonal anatomy. FA is influenced by fiber orientation, crossing fibers, and the relative contribution of different fiber populations and therefore does not directly represent axonal integrity or density [14]. In glioma, tumor infiltration and vasogenic edema may further alter FA by changing tissue water content and fiber organization. Tractography-derived length and volume likewise depend on successful streamline reconstruction under pathological conditions. Comparisons with intraoperative stimulation have shown that fiber tracking may not accurately represent the anatomical extent of a fiber bundle [15], and deterministic DTI is limited in regions with crossing fibers or altered diffusion properties in brain tumors [16]. Tract length was therefore included as a descriptor of the reconstructed pathway rather than as a measure of anatomical shortening or elongation of the CST.

Tracking threshold also affects the extent of reconstructed fibers. In glioma patients, increasing the FA threshold can progressively reduce tract reconstruction and prevent visualization of fibers traversing tumor-infiltrated regions [17]. Wende et al. proposed an FA cutoff around 0.15 as a practical starting point for CST tractography, while noting that lower thresholds may be required when anisotropy is reduced [18]. The similar corrected findings at FAT 0.10 and 0.15 support the robustness of the main quantitative inference, whereas the uncorrected volume difference observed only at FAT 0.15 illustrates sensitivity to threshold selection.

### Contralesional CST relative to the HCP842 normative reference

Both primary and recurrent contralesional CST trajectories were posterior to the HCP842 reference, whereas the recurrent trajectory was less posterior, consistent with the paired anterior displacement. HCP842 therefore served as a fixed spatial reference for contextualizing the longitudinal change rather than as an independent healthy-control comparison.

HCP842 is a population-averaged structural connectome derived from diffusion MRI data from 842 Human Connectome Project participants [11]. However, CST anatomy varies considerably between individuals, particularly toward its cortical projections [19], and DTI-based tractography shows incomplete spatial reproducibility across repeated acquisitions [20]. In addition, HCP842 and the present CST reconstructions were generated using different diffusion acquisition and tractography pipelines. Absolute patient-to-atlas offsets should therefore be interpreted descriptively rather than as direct measures of abnormal CST anatomy.

### Clinical and methodological implications

DTI-based CST tractography is widely used to characterize the spatial relationship between motor pathways and gliomas during preoperative assessment [2, 3]. Because contralesional CST position differed between the primary and recurrent assessments within the same patients, longitudinal evaluation should use contemporaneous tractography when available rather than assume that earlier spatial information remains unchanged.

The contralesional CST remains a useful patient-specific comparator, but its spatial position should not be assumed to remain fixed in longitudinal analyses. These spatial changes occurred despite MRC 5/5 strength at both assessments; therefore, their functional significance cannot be determined from the present data. Future prospective studies incorporating quantitative motor and fine-motor testing could examine whether the magnitude and direction of CST displacement are associated with motor performance, tumor proximity, or intervening treatment exposure, and whether recurrence-stage tractography better reflects contemporaneous CST position than earlier tractography.

### Limitations

Several limitations should be acknowledged. First, this retrospective single-center study included 29 patients with paired preoperative DTI, successful CST reconstruction and normalization, and MRC 5/5 strength at both assessments, limiting generalizability to patients with greater neurological impairment or technically inadequate tractography. Requiring paired preoperative DTI also selected patients who survived to recurrence and remained eligible for repeat imaging, introducing potential selection bias. The sample size limited subgroup analyses according to tumor proximity and molecular characteristics. Detailed voxel-wise modeling of tumor spatial distribution was not performed, and tumor-to-ipsilateral-CST distance was not quantified longitudinally; therefore, the contribution of regional tumor location and local tumor–tract proximity remains unresolved. Surgery and adjuvant treatment occurred between assessments in most patients, so the observed changes cannot be attributed to recurrence alone. Detailed radiotherapy fraction-level data and exact treatment-to-imaging intervals were unavailable from historical records. Quantitative motor and fine-motor testing was also unavailable, limiting assessment of functional relevance.

Second, deterministic DTI tractography is sensitive to crossing fibers, altered diffusion, ROI definition, and tracking thresholds. Although the same anatomical criteria and tracking parameters were applied at both assessments and spatial normalization underwent visual quality control, reconstructed trajectories remain method-dependent, and small spatial differences may partly reflect residual normalization uncertainty. HCP842 comparisons were descriptive because the atlas was generated using a different diffusion acquisition and tractography pipeline. The exploratory bilateral analysis was additionally limited by its small sample (*n* = 13) and different cortical seeding strategies between sides.

## Conclusion

In this within-patient longitudinal study, the contralesional CST showed a predominantly anterior shift between the primary and recurrent assessments, whereas FA, tract length, and tract volume showed no significant longitudinal differences after correction for multiple comparisons. Relative to HCP842, the recurrent CST was less posterior than at the primary assessment. These findings indicate that contralesional CST position may change over time and should not be assumed to remain spatially fixed in longitudinal glioma studies.

## Supplementary Information

Below is the link to the electronic supplementary material.

Supplementary Material 1

## Data Availability

The datasets used and analyzed during the current study are available from the corresponding author upon reasonable request.

## Abbreviations

CST: Corticospinal tract
DTI: Diffusion tensor imaging
FA: Fractional anisotropy
FAT: Fractional anisotropy threshold
nTMS: Navigated transcranial magnetic stimulation
ROI: Region of interest

## References

[1] Aftahy AK, Groll M, Wagner A, Barz M, Bernhardt D, Combs SE, Meyer B, Gempt J, Negwer C (2022) Schwannomas of the greater superficial petrosal nerve - case series, discussion of surgical techniques, and review of literature. BMC Neurol 22:470. 10.1186/s12883-022-02960-3 PMC9733176 36494617

[2] Krieg SM, Sabih J, Bulubasova L, Obermueller T, Negwer C, Janssen I, Shiban E, Meyer B, Ringel F (2014) Preoperative motor mapping by navigated transcranial magnetic brain stimulation improves outcome for motor eloquent lesions. Neurooncology 16:1274–1282. 10.1093/neuonc/nou007 PMC4136889 24516237

[3] Weiss Lucas C, Faymonville AM, Loução R, Schroeter C, Nettekoven C, Oros-Peusquens AM, Langen KJ, Shah NJ, Stoffels G, Neuschmelting V, Blau T, Neuschmelting H, Hellmich M, Kocher M, Grefkes C, Goldbrunner R (2022) Surgery of Motor Eloquent Glioblastoma Guided by TMS-Informed Tractography: Driving Resection Completeness Towards Prolonged Survival. Front Oncol 12:874631. 10.3389/fonc.2022.874631 PMC9186060 35692752

[4] Zhylka A, Sollmann N, Kofler F, Radwan A, De Luca A, Gempt J, Wiestler B, Menze B, Schroeder A, Zimmer C, Kirschke JS, Sunaert S, Leemans A, Krieg SM, Pluim J (2023) Reconstruction of the Corticospinal Tract in Patients with Motor-Eloquent High-Grade Gliomas Using Multilevel Fiber Tractography Combined with Functional Motor Cortex Mapping. AJNR Am J Neuroradiol 44:283–290. 10.3174/ajnr.A7793 PMC10187805 36797033

[5] Cepeda S, García-García S, Arrese I, Velasco-Casares M, Sarabia R (2021) Acute changes in diffusion tensor-derived metrics and its correlation with the motor outcome in gliomas adjacent to the corticospinal tract. Surg Neurol Int 12:51. 10.25259/sni_862_2020 PMC7911041 33654554

[6] Weiss C, Tursunova I, Neuschmelting V, Lockau H, Nettekoven C, Oros-Peusquens AM, Stoffels G, Rehme AK, Faymonville AM, Shah NJ, Langen KJ, Goldbrunner R, Grefkes C (2015) Improved nTMS- and DTI-derived CST tractography through anatomical ROI seeding on anterior pontine level compared to internal capsule. NeuroImage Clin 7:424–437. 10.1016/j.nicl.2015.01.006 PMC4314616 25685709

[7] Genç B, Aslan K, Özçağlayan A, İncesu L (2024) Microstructural abnormalities in the contralateral normal-appearing white matter of glioblastoma patients evaluated with advanced diffusion imaging. Magnetic resonance in medical sciences: MRMS : an official journal of Japan Society of Magnetic Resonance in Medicine. 23:479–486. 10.2463/mrms.mp.2023-0054 PMC11447469 37532585

[8] Kallenberg K, Goldmann T, Menke J, Strik H, Bock HC, Mohr A, Buhk JH, Frahm J, Dechent P, Knauth M (2014) Abnormalities in the normal appearing white matter of the cerebral hemisphere contralateral to a malignant brain tumor detected by diffusion tensor imaging. Folia Neuropathol 52:226–233. 10.5114/fn.2014.45563 25310733

[9] Hope TR, Vardal J, Bjørnerud A, Larsson C, Arnesen MR, Salo RA, Groote IR (2015) Serial diffusion tensor imaging for early detection of radiation-induced injuries to normal-appearing white matter in high-grade glioma patients. J Magn Reson imaging: JMRI 41:414–423. 10.1002/jmri.24533 24399480

[10] Rydelius A, Lampinen B, Rundcrantz A, Bengzon J, Engelholm S, van Westen D, Kinhult S, Knutsson L, Lätt J, Nilsson M, Sundgren PC (2022) Diffusion tensor imaging in glioblastoma patients treated with volumetric modulated arc radiotherapy: a longitudinal study. Acta Oncol (Stockholm Sweden) 61:680–687. 10.1080/0284186x.2022.2045036 35275512

[11] Yeh FC, Panesar S, Fernandes D, Meola A, Yoshino M, Fernandez-Miranda JC, Vettel JM, Verstynen T (2018) Population-averaged atlas of the macroscale human structural connectome and its network topology. NeuroImage 178:57–68. 10.1016/j.neuroimage.2018.05.027 PMC6921501 29758339

[12] Field AS, Alexander AL, Wu YC, Hasan KM, Witwer B, Badie B (2004) Diffusion tensor eigenvector directional color imaging patterns in the evaluation of cerebral white matter tracts altered by tumor. J Magn Reson imaging: JMRI 20:555–562. 10.1002/jmri.20169 15390227

[13] Mahmoodi AL, Landers MJF, Rutten GM, Brouwers HB (2023) Characterization and classification of spatial white matter tract alteration patterns in glioma patients using magnetic resonance tractography: a systematic review and meta-analysis. Cancers 15. 10.3390/cancers15143631 PMC10377290 37509291

[14] Figley CR, Uddin MN, Wong K, Kornelsen J, Puig J, Figley TD (2021) Potential Pitfalls of Using Fractional Anisotropy, Axial Diffusivity, and Radial Diffusivity as Biomarkers of Cerebral White Matter Microstructure. Front NeuroSci 15:799576. 10.3389/fnins.2021.799576 PMC8795606 35095400

[15] Kinoshita M, Yamada K, Hashimoto N, Kato A, Izumoto S, Baba T, Maruno M, Nishimura T, Yoshimine T (2005) Fiber-tracking does not accurately estimate size of fiber bundle in pathological condition: initial neurosurgical experience using neuronavigation and subcortical white matter stimulation. NeuroImage 25:424–429. 10.1016/j.neuroimage.2004.07.076 15784421

[16] Bucci M, Mandelli ML, Berman JI, Amirbekian B, Nguyen C, Berger MS, Henry RG (2013) Quantifying diffusion MRI tractography of the corticospinal tract in brain tumors with deterministic and probabilistic methods. NeuroImage Clin 3:361–368. 10.1016/j.nicl.2013.08.008 PMC3815019 24273719

[17] Stadlbauer A, Nimsky C, Buslei R, Salomonowitz E, Hammen T, Buchfelder M, Moser E, Ernst-Stecken A, Ganslandt O (2007) Diffusion tensor imaging and optimized fiber tracking in glioma patients: Histopathologic evaluation of tumor-invaded white matter structures. NeuroImage 34:949–956. 10.1016/j.neuroimage.2006.08.051 17166744

[18] Wende T, Kasper J, Wilhelmy F, Dietel E, Hamerla G, Scherlach C, Meixensberger J, Fehrenbach MK (2021) Assessment of a reliable fractional anisotropy cutoff in tractography of the corticospinal tract for neurosurgical patients. Brain Sci 11. 10.3390/brainsci11050650 PMC8155834 34065682

[19] Dalamagkas K, Tsintou M, Rathi Y, O’Donnell LJ, Pasternak O, Gong X, Zhu A, Savadjiev P, Papadimitriou GM, Kubicki M, Yeterian EH, Makris N (2020) Individual variations of the human corticospinal tract and its hand-related motor fibers using diffusion MRI tractography. Brain imaging Behav 14:696–714. 10.1007/s11682-018-0006-y PMC6614022 30617788

[20] Kristo G, Leemans A, de Gelder B, Raemaekers M, Rutten GJ, Ramsey N (2013) Reliability of the corticospinal tract and arcuate fasciculus reconstructed with DTI-based tractography: implications for clinical practice. Eur Radiol 23:28–36. 10.1007/s00330-012-2589-9 22868481

