# Supplementary material for: Longitudinal spatial changes of the contralesional corticospinal tract in high-grade glioma: a within-patient DTI tractography study
Source: J Neurooncol. 2026 Oct 9;180(1):10. doi: 10.1007/s11060-026-05823-w (PMC13652912; doi:10.1007/s11060-026-05823-w)
Supplement: Supplementary file 1 [file 11060_2026_5823_MOESM1_ESM.docx]

**Supplementary Material**

**Supplementary Results**

**Supplementary voxel-wise analysis**

Paired voxel-wise analysis was performed using exact McNemar tests across the 29 matched primary and recurrent assessments. At both FAT 0.10 and FAT 0.15, no voxel remained significant after either BH-FDR or Bonferroni correction. Uncorrected P < 0.05 maps after a minimum cluster-size filter of 20 contiguous voxels showed spatially heterogeneous regions in which CST occupancy was greater at either the primary or recurrent assessment (Supplementary Fig. S1). These maps were considered exploratory and were not interpreted as statistically corrected spatial differences.

**Supplementary Fig. S1. Paired voxel-wise spatial analysis of the contralesional CST**

**(a, b)** FAT 0.10 and **(c, d)** FAT 0.15 analyses. Panels a and c show voxel-wise paired frequency differences between the recurrent and primary assessments across the 29 matched patients. Positive values indicate greater CST occupancy at recurrence, whereas negative values indicate greater occupancy at the primary assessment. Panels b and d show voxels with uncorrected exact McNemar *P* < 0.05 after application of a minimum cluster-size filter of 20 contiguous voxels. Blue indicates greater occupancy at the primary assessment and orange greater occupancy at recurrence. No voxel survived BH-FDR or Bonferroni correction at either FAT; therefore, the cluster-filtered maps are exploratory and descriptive only.

**Individual longitudinal variability in CST displacement**

Individual recurrence-minus-primary centroid differences demonstrated substantial heterogeneity across patients and axial levels (Supplementary Fig. S2). Changes along the Y axis showed a predominantly anterior direction in many patients, consistent with the group-level findings, but both the magnitude and direction of displacement varied between individuals. X-axis changes were generally smaller and showed a more mixed mediolateral pattern. Thus, the group-level anterior displacement represented an average tendency superimposed on substantial patient-specific spatial variability.

**Supplementary Fig. S2. Individual longitudinal heterogeneity in contralesional CST displacement**

Patient-level recurrence-minus-primary centroid differences across MNI Z levels at **(a, b)** FAT 0.10 and **(c, d)** FAT 0.15. Panels a and c show mediolateral displacement (ΔX), with positive values indicating medial displacement and negative values indicating lateral displacement. Panels b and d show anteroposterior displacement (ΔY), with positive values indicating anterior displacement and negative values indicating posterior displacement. Each row represents one of the 29 paired patients. The heatmaps demonstrate substantial interpatient variation in the magnitude and direction of longitudinal CST displacement, particularly despite the predominantly anterior group-level Y-axis shift.

**Exploratory bilateral analysis**

The exploratory bilateral analysis included 13 patients with both tumor-side and contralesional CST masks available at the primary and recurrent assessments and at both FATs (Supplementary Fig. S3). No side-by-time interaction remained significant after BH-FDR correction across the global bilateral tests. At FAT 0.15, the tumor-side CST was located anterior to the contralesional CST at the recurrent assessment, with a mean tumor-side-minus-contralesional Y difference of 4.453 mm (95% CI, 2.077–6.830; q = 0.009). Because the tumor-side CST was reconstructed using nTMS-based cortical seeding whereas the contralesional CST used anatomical cortical seeding, the bilateral analysis was considered exploratory and was not used to infer biological side-specific remodeling.

**Supplementary Fig. S3. Exploratory bilateral CST trajectories at the primary and recurrent assessments**

Mean tumor-side and contralesional CST centroid trajectories in the subset of 13 patients with bilateral CST masks available at both assessments. **(a–d)** FAT 0.10 and **(e–h)** FAT 0.15. X-axis trajectories are shown in panels a, c, e, and g, and Y-axis trajectories in panels b, d, f, and h. Lines represent patient-level mean trajectories and shaded areas indicate 95% confidence intervals. Tumor-side CSTs were reconstructed using nTMS-based motor cortical seeding, whereas contralesional CSTs used anatomical cortical seeding. Accordingly, bilateral comparisons were exploratory. No side-by-time interaction survived BH-FDR correction across the global bilateral tests.

**Mediolateral CST position relative to HCP842**

Descriptive comparison with the HCP842 normative CST reference showed relatively small patient-minus-reference offsets along the X axis (Supplementary Fig. S4). At FAT 0.10, the mean X offsets were +2.00 mm at the primary assessment and +2.45 mm at recurrence. At FAT 0.15, the corresponding offsets were +2.28 and +2.41 mm. Positive values indicate a more medial position relative to the HCP842 reference. These comparisons were descriptive because HCP842 represents a fixed normative reference generated using a different diffusion acquisition and tractography pipeline rather than an independent healthy-control cohort.

**Supplementary Fig. S4. Mediolateral position of the contralesional CST relative to the HCP842 normative reference**

(a, b) FAT 0.10 and (c, d) FAT 0.15 analyses. Panels a and c show mean X-centroid trajectories of the primary and recurrent contralesional CSTs together with the fixed HCP842 normative CST reference. Panels b and d show the corresponding patient-minus-HCP842 X offsets across MNI Z levels. Positive offsets indicate a more medial position and negative offsets a more lateral position relative to HCP842. Shaded areas indicate 95% confidence intervals for the patient trajectories, and the dashed vertical line denotes zero offset. Mean X offsets were +2.00 mm and +2.45 mm at FAT 0.10 and +2.28 mm and +2.41 mm at FAT 0.15 for the primary and recurrent assessments, respectively. Comparisons with HCP842 were descriptive because the atlas represents a fixed normative reference rather than an independent healthy-control cohort.

**Paired individual distributions of tractography-derived CST parameters**

Individual paired observations showed substantial interpatient variability in mean FA, tract length, and tract volume at both FATs (Supplementary Fig. S5). The primary and recurrent distributions showed considerable overlap for all six comparisons, with no consistent directional change in mean FA or tract length. At FAT 0.15, tract volume was lower at recurrence at the group level before multiple-comparison correction, but this difference did not remain significant after BH-FDR correction. Overall, the individual-level distributions were consistent with the group-level inference that none of the six tractography-derived parameter comparisons remained significant after correction.

**Supplementary Fig. S5. Paired individual longitudinal comparisons of contralesional CST tractography-derived parameters.**
Paired primary and recurrent values are shown for mean FA, tract length, and tract volume at FAT 0.10 (a, c, e) and FAT 0.15 (b, d, f) in the 29 matched patients. Each gray line connects the primary and recurrent measurements from the same patient; colored points indicate individual observations. Diamonds and error bars indicate the group mean and 95% confidence interval. P values are from paired t tests, q values are BH-FDR-corrected across the six prespecified longitudinal comparisons, and Cohen’s dz represents the paired-sample effect size. None of the six comparisons remained significant after BH-FDR correction.
